# Behavioural heterogeneity across killer whale social units in their response to feeding opportunities from fisheries

**DOI:** 10.1002/ece3.11448

**Published:** 2024-05-23

**Authors:** Erwan Auguin, Christophe Guinet, Johann Mourier, Eric Clua, Nicolas Gasco, Paul Tixier

**Affiliations:** ^1^ UMR MARBEC Université de Montpellier‐CNRS‐IFREMER‐IRD Sète France; ^2^ Centre d'Etudes Biologiques de Chizé (CEBC) UMR 7372 CNRS‐La Rochelle Université – CNRS Villiers‐en‐Bois France; ^3^ Université Paris Sciences & Lettres (PSL), CRIOBE USR3278, EPHE‐CNRS‐UPVD Université de Perpignan Perpignan France; ^4^ Laboratoire D'Excellence LabEX CORAIL CRIOBE, Baie Opunohu Papetoai French Polynesia; ^5^ Laboratoire de Biologie Des Organismes et Ecosystèmes Aquatiques (BOREA) UMR 8067 – MNHN, CNRS, IRD, Su, UCN, UA Paris France

**Keywords:** depredation, human‐wildlife conflicts, intra‐population variation, marine top predator, *Orcinus orca*, social network

## Abstract

Intra‐population heterogeneity in the behavioural response of predators to changes in prey availability caused by human activities can have major evolutionary implications. Among these activities, fisheries, while extracting resources, also provide new feeding opportunities for marine top predators. However, heterogeneity in the extent to which individuals have responded to these opportunities within populations is poorly understood. Here, we used 18 years of photo‐identification data paired with statistical models to assess variation in the way killer whale social units within a subantarctic population (Crozet Islands) interact with fisheries to feed on fish caught on fishing gear (i.e., depredation behaviour). Our results indicate large heterogeneity in both the spatial and temporal extents of depredation across social units. While some frequently depredated on fishery catches over large areas, others sporadically did so and in small areas consistently over the years. These findings suggest that killer whale social units are exposed to varying levels of impacts of depredation, both negative (potential retaliation from fishers) and positive (food provisioning), on their life history traits, and may explain the contrasted demographic patterns observed within the declining population at Crozet but also potentially within the many other killer whale populations documented depredating on fisheries catches worldwide.

## Graphical abstract

We used a unique long‐term monitoring dataset of a subantarctic killer whale population, spanning over 18 years, paired with statistical models, to assess variation in the way killer whale social units depredate fish catches on fishing gear in a commercial fishery that started in the 1990s. Our results indicate large heterogeneity in both the spatial and temporal extents of depredation across social units. While some frequently interacted with fishing vessels over large areas, others sporadically did so and in small areas, consistently over the years. These findings suggest that killer whale social units are exposed to varying levels of impacts of depredation.

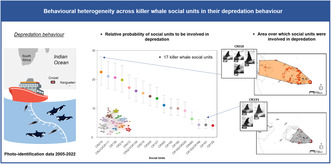

## 1 INTRODUCTION

Behavioural heterogeneity within animal populations occurs when individuals, or groups of individuals in social species, show variability in specific behaviours such as foraging (Farine et al., 2015; Jolles et al., 2020; Kaufhold & van Leeuwen, 2019; Planas‐Sitjà et al., 2015). By leading to contrasted fitness performances across individuals, such heterogeneity is a key determinant of population dynamics and species evolutionary trajectories (Bolnick et al., 2011; Hart et al., 2016; Vindenes & Langangen, 2015). It can be driven by multiple factors such as intra‐ and interspecific competition, personality traits of individuals and/or variation in habitat and resource availability (Araújo et al., 2011; Bolnick et al., 2003; Dall et al., 2012; Montiglio et al., 2013; Svanbäck & Bolnick, 2006). In particular, the incidence of the latter was highlighted by a strong intra‐population heterogeneity observed in the behavioural response to human‐induced changes in resource availability, such as the emergence of new feeding opportunities in the form of anthropogenic subsidies (Larson et al., 2020; Oro et al., 2013; Sanz‐Aguilar et al., 2015; West & Jones, 2022).

In the marine environment, fisheries are among human activities that have most profoundly altered ecosystems in the world's oceans over the past 60 years (Pauly et al., 2005). Although fisheries decrease prey availability for predators through resource extraction, they can also provide feeding opportunities to these species in the form of fish discards (Le Bot et al., 2018) or fish caught on fishing gear (Mitchell et al., 2018; Tixier, Lea, et al., 2021). Many species of seabirds, sharks and marine mammals have been documented exploiting these opportunities, but little is known about how new foraging behaviours associated with fishing activities may have been developed to varying extents by individuals within populations. Yet, these behaviours can impact the fitness of individuals either negatively (injury or death from interaction with the gear or retaliation practices from fishers; Lewison et al., 2004; Tixier et al., 2017) or positively (access to prey at low foraging effort; Tixier et al., 2015), or both. Therefore, quantifying and understanding individual heterogeneity in this behaviour is essential to assess its effects on the populations involved and the consequences of feeding on fisheries subsidies on ecosystems. These include changes in predation pressures from marine predators on natural prey and the subsequent alteration of trophic interactions (Newsome et al., 2015).

The killer whale *Orcinus orca* is one of the marine top predator species most frequently reported feeding on fisheries catches on fishing gear (Tixier, Lea, et al., 2021). This behaviour, termed “depredation,” has been documented in many killer whale populations around the world, especially in fisheries using longlines (lines bearing series of baited hooks; Bearzi et al., 2019; Hamer et al., 2012). As a highly social species, killer whales generally depredate in groups of closely related individuals (hereafter “social units” – in which stability varies across populations; Baird & Dill, 1996; Ford, 2019). Although heterogeneity across social units has been demonstrated within multiple populations in regard to their foraging behaviours on natural prey (Jourdain et al., 2020; Reisinger et al., 2016; Samarra et al., 2017), evidence of variation in the extent to which social units have developed the depredation behaviour in response to fisheries is still lacking. As killer whales are long‐lived top predators, such heterogeneity, by exposing social units to varying levels of impacts from depredation on fisheries catches, may lead to divergent demographic trajectories within populations, with potentially strong evolutionary, ecological and conservation implications in the long term.

Around the Crozet Islands (subantarctic islands in the Indian Ocean sector of the Southern Ocean), the extent of killer whale depredation is among the highest across all cases of killer whale depredation worldwide (Tixier, Lea, et al., 2021). Individuals feed on Patagonian toothfish *Dissostichus eleginoides* caught on longlines at a rate of >40% of all longlines deployed by the 8 licensed vessels of the commercial fishery and remove 179 tons of fish per year (21.4% of the total catch; Tixier et al., 2020). Two genetically and ecologically segregated forms of killer whales are involved in these interactions: the so‐called “Crozet killer whales” and “Type‐D killer whales” (Tixier et al., 2016). Type‐D killer whales are only sighted in offshore waters and have been identified sporadically around fishing vessels in Crozet waters since 2003 (Tixier et al., 2016). The Crozet killer whales dominate the depredation events, are encountered in both offshore and inshore waters, and are generalist in their feeding preferences, with prey including seals, penguins, large whales and fish (Tixier et al., 2019). Individuals from this form have been monitored through photo‐identification since the 1960s, and these data indicate a sharp decline of the population in the 1990s. This was mainly caused by individuals being shot when depredating around the many fishing vessels operating illegally around the islands between 1996 and 2003 (Guinet et al., 2015). The population has continued declining in the 2000s and 2010s and is now reduced to 80–90 individuals (Tixier, Gasco, et al., 2021). Factors explaining this prolonged decline likely include deep changes in the social organisation caused by the over‐mortality of the 1990s (Busson et al., 2019) and individuals being still exposed to lethal practices in areas where illegal fishing persists (Tixier, Gasco, et al., 2021). For these reasons, understanding whether some social units of the Crozet killer whales are more involved in depredation, and thus more exposed to the potential impacts of this behaviour on their survival, than others, has become critical for the conservation of the population.

Therefore, in this study, using killer whale photo‐identification data and fishing data collected around the Crozet Islands over an 18‐year period (2005–2022), we aimed to investigate heterogeneity in the extent to which social units have responded to opportunities to feed on fish caught on fishing gear. Specifically, our goal was to (i) identify the social units involved in the depredation of toothfish caught by the longline fishery and (ii) assess variation between social units in their spatio‐temporal occurrence during depredation events.

## 2 MATERIALS AND METHODS

### 2.1 Data collection

We used photographic identification data of the Crozet killer whales collected by trained personnel between 2005 and 2022 from two platforms: from the shore of Possession Island (46°S–51°E) by fieldworkers when individuals foraged on seals and penguins along the coast (Tixier et al., 2019) and from all licensed fishing vessels (i.e., from seven to eight vessels per year over the period) of the longline toothfish fishery by fishery observers in the Crozet Exclusive Economic Zone (EEZ, 44–48°S–45–55°E). Fishery observers were present on these longliners at all times and monitored 100% of the fishing operations. They recorded information on the date, time, location, fishing effort and catch, as well as on the occurrence of whale depredation, for all longline sets (a longline set is made of a main line of three to seven kilometres long, bearing thousands of baited hooks deployed on the seafloor at depths >500 m for hours before being hauled back onto the vessel). Fishery observers confirmed the occurrence of killer whale depredation during the hauling of a given longline set (hereafter referred to as a “depredation event”) using a combination of cues visible from the surface: (i) killer whales were observed foraging over prolonged periods of time (from one to several hours) within a 500 m radius around the vessel by repeatedly diving towards the longline being hauled; (ii) killer whale individuals were surrounded by seabirds and fish oil slicks when surfacing; (iii) fish heads or lips were present on the hooks on the longline hauled while killer whales were around (Tixier et al., 2010).

We assigned a unique code to each killer whale sighting during which photographs were taken. We defined a sighting (i) spatially as a set of photographs taken from the same site, i.e., from fishing vessels: during the hauling of one longline set by one fishing vessel and from the shore: in one of the bays of the island and (ii) temporally as the time elapsed from the first to the last photograph taken by the observer, i.e., from fishing vessels: as the set of photographs taken during the hauling of one longline set by one fishing vessel, and from the shore: as the set of photographs taken with <1 h of time interval between two consecutive photographs. For sightings from fishing vessels, we only used photographs taken during depredation events (i.e., only photographs of killer whales depredating fish on longlines).

Date, time and location data were recorded for each sighting. For sightings from fishing vessels, these data also included the identity of the vessel and were extracted from the “PECHEKER” database of the Muséum National d'Histoire Naturelle (Martin et al., 2021). Killer whale individuals were identified based on the natural markings of their dorsal fin and saddle patch (Bigg et al., 1987; Tixier, Gasco, et al., 2021). Data on the ID of individuals were recorded for each photograph through a frame‐by‐frame analysis. In all subsequent analyses, we used the number of photographs taken during sightings as the metric for the photographic effort, which was shown to positively influence the number of killer whale individuals identified during sightings at Crozet (Tixier et al., 2017; Tixier, Gasco, et al., 2021).

### 2.2 Characterisation of social units

We performed a social network analysis in R (R Core Team, 2023) with the packages “asnipe” (Farine, 2013) and “igraph” (Csardi & Nepusz, 2006) using the photo‐identification data collected both from the shore and from the fishing vessels to measure associations between individuals and determine the social units composing the Crozet killer whale population. Our approach involved several successive steps: (i) building the association matrix from the frequency of co‐occurrences between individuals, (ii) testing for the presence of preferred associations in the network by comparing the real association matrix to a null model, and (iii) characterising social units with a community detection algorithm to determine significant clusters in which members are more associated together than they are with other individuals outside the cluster.

In order to reduce bias associated with these data (Whitehead, 2008a), we restricted the dataset to (i) juveniles and adults that were photographed over at least 6 years between 2005 and 2022, and which were last photographed after 2019, and (ii) sightings with a photographic effort being high enough to assume that all the individuals present were photographed. In this study, because of the large number of sightings we had in hand, we chose a conservative approach based on Ottensmeyer and Whitehead (2003) by selecting sightings with a number of photographs at least three times greater than the mean number of individuals identified per sighting (8.2 ± 5.7 SD (standard deviation) individuals (*n* = 1121 sightings)) over the period 2005–2022. This threshold was set to 42 photographs (from a dataset ranging from 1 to 3299 photographs per sighting), which also corresponded to the minimum number of photographs taken during 50% of the sightings over the study period. The restricted dataset included 79 killer whale individuals with photo‐identification information from a total of 180,664 photographs taken during 1121 sightings (168 from the shore, 953 from fishing vessels) made over 760 days (140 from the shore, 630 from fishing vessels). The duration of sightings was not significantly different between sightings from the shore and from fishing vessels (Wilcoxon‐Mann–Whitney test, *p*‐value >.05), with a mean of 39 ± 3 SE (Standard Error) min per sighting from the shore (*n* = 168 sightings) and 39 ± 2 SE min per sighting from fishing vessels (*n* = 953 sightings).

First, we used the Simple Ratio Index (SRI; Cairns & Schwager, 1987) to assess the strength of association between individuals. We considered two individuals as associated during a sighting if photographed during the same sighting. This index is calculated as an estimate of the proportion of time two animals spend together (“0” for pairs of animals never observed together – “1” for pairs of animals always observed together). It does not overestimate associations between individuals and is the most appropriate when assumption into observation errors cannot be accounted for (Hoppitt & Farine, 2018), such as, in our case, associations that we could not observe as occurring away from the observer. In practice, groups (defined in our analysis as sets of individuals photographed during the same sighting) were clearly identifiable because sightings were spatio‐temporally well defined. Our sampling method involved taking the “gambit of the group” (Whitehead & Dufault, 1999), assuming that all individuals present in a group together were associated. From our large dataset, we conducted a Mantel Test (Sperman's rank correlation – 10,000 permutations) to check for statistical differences between associations of killer whale individuals as assessed using photographic data collected from two platforms (fishing vessels and the shore of Possession Island). This test allowed us to check for potential bias associated with preferential associations being significantly different between situations when killer whales were sighted around fishing vessels and around Possession Island.

We calculated the coefficient of variation of the SRI (S) and the correlation coefficient of the true and estimated association matrices (r) using maximum likelihood procedures (Whitehead, 2008b). S is a measure of social differentiation in a population and r is a measure of the power of the analysis to detect the true pattern of social structure (Appendix S1: Supporting text). We used permutation tests (10,000 permutations with 10 trials per permutation) to assess whether individuals associated randomly or had preferred/avoided associations (Bejder et al., 1998; Manly, 1995; Whitehead et al., 2005), by permuting daily association data (individuals were considered associated if sighted together during the same day). Preferred associations are expected if the SD or CV (coefficient of variation) of associations of the observed network are higher than the SD or CV measured from the 10,000 randomised versions of the network. The result is significant at *p*‐value = .05 if fewer than 2.5% of the random values of SD (or CV) are greater than the observed value of SD (or CV).

Social units were here defined as groups of killer whale individuals characterised by strong and long‐term associations. We used the Leiden algorithm based on Constant Potts Model with a gamma resolution parameter (γ) and 20,000 iterations to detect and define social units within the association network (Arenas et al., 2008; Traag et al., 2019). This approach relies on densely connected individuals within the association network, assuming these individuals are more strongly associated with each other than with others, to delineate social units. Contrary to the community detection algorithm based on modularity, the significance of partitions in the social units derived from the Leiden algorithm does not need to be checked using a null model (Traag et al., 2011).

### 2.3 Comparison of the extent of depredation between social units

We assessed heterogeneity in the depredation behaviour of killer whale social units, as identified from the social network analysis, using the photo‐identification information collected from the fishing vessels (160,662 usable killer whale photographs taken during 1475 sightings between 2005 and 2022). These photographs were available for a subset of all killer whale depredation events, i.e., 30% of all killer whale depredation events recorded from licensed fishing vessels by fishery observers, with the possibility that killer whale depredation events also occurred around fishing vessels operating illegally in the area, although illegal fishing was greatly reduced past 2003. Therefore, we examined this heterogeneity as the relative variation in the extent to which a killer whale social unit was involved in depredation events in comparison with others.

Firstly, we compared the spatial range over which social units were sighted while depredating toothfish on longlines, assuming that when at least one individual from a given unit was photographed during a sighting, the whole social unit was present during that sighting. We used three approaches to estimate this range from the location of sightings: (i) the minimum convex polygons (MCPs) in km^2^ as a simple representation of the full home range of animal species (Mohr, 1947), (ii) the kernel density estimation (KDE) to measure the utilisation distribution (UD) in km^2^ at 50% (UD50) and 95% (UD95) (Worton, 1989), and (iii) the number of 0.1° × 0.1° (10 km × 10 km) cells in which social units were sighted during depredation events over a spatial grid. For both the MCP and the spatial grid approaches, we calculated the proportion of the fishing area over which social units were sighted during depredation events as the ratio between the MCP or the number of spatial cells in which a given social unit was sighted and the MCP or the number of spatial cells in which any social unit was sighted (hereafter “fishing area”). We calculated this proportion (i) using the total area in which social units were sighted over the whole study period and (ii) using the cumulative area in which social units were sighted per year, and over the years following the year social units were first sighted, to examine variation in the extent to which social units expanded their spatial range of depredation over time. Potential correlations between the total spatial range of depredation events during which social units were sighted and the number of years social units were sighted were examined using Spearman's test for non‐parametric data.

Secondly, we used a modelling approach to investigate variation in the relative probability of occurrence of killer whale social units during depredation events (the probability of a given social unit to be present during depredation events relative to the other social units). To limit the potential overestimation of probabilities due to killer whale social units, once they have found a fishing vessel, generally following it and depredating on longlines it hauls consecutively (Cieslak et al., 2021), for this analysis we excluded sightings of the same social unit within 12 h of their first sighting around the same fishing vessel. This restricted the data used for the model to 1212 sightings (82% of all sightings). From this dataset, we used a generalised linear mixed model (GLMM) with the “glmmTMB” package in R (Brooks et al., 2017), using a binomial distribution and a logit link function (Bolker et al., 2009; Zuur et al., 2009). The response variable was the occurrence of social units during sightings. For each sighting, multiple records were created, one for each social unit, with a binary response variable indicating presence (1) or absence (0) of the unit during the sighting. The model incorporated the ID of the social unit, the year and the month as fixed terms and the photographic effort as a random term. The photographic effort was incorporated as a five‐level categorical term to account for the positive influence of the number of photographs taken on the number of killer whale individuals photographed, and therefore the probability to detect the social units present, during sightings, with levels being: very low (<25 photographs), low (≥25 and <50 photographs), medium (≥50 and <125 photographs), high (≥125 and <250 photographs) and very high (≥250 photographs). Given the hierarchical structure of the data we used for the model, we addressed potential pseudoreplication effects by testing an alternative model with the social unit ID nested into the sighting as a random term. We fitted the model using the “optim” optimiser with the “L‐BFGS‐B” method, which improved model convergence. We selected the final model using a stepwise forward selection based on the Akaike's Information Criterion corrected for small samples (AICc) using the R package “MuMIn” (Bartoń, 2023). We assessed the goodness‐of‐fit of the final model by calculating the conditional and marginal coefficients of determination *R* ^2^ for GLMMs (Nakagawa & Schielzeth, 2013) and we calculated *p*‐values using the Anova function of the “car” package in R (Fox & Weisberg, 2019) after checking the homogeneity of variances (Bolker et al., 2009). We validated and checked model assumptions by following the DHARMa protocol (Hartig, 2022), simulating 1000 datasets from the fitted model to test if the distribution of the scaled residuals deviated from the expected distribution. The relative probability of each social unit to be present during depredation events was predicted from the model outputs and we used the pairwise comparisons of Estimated Marginal Means (EMMs, method “Holm” – package “emmeans” in R (Lenth, 2023)) as a post hoc test of differences in the probability between units. We conducted all analyses using R 4.3.0 (R Core Team, 2023).

## 3 RESULTS

### 3.1 Characterisation of social units

From the photo‐identification data collected on 79 killer whale individuals during 1121 sightings between 2005 and 2022, the correlation between the associations among killer whale individuals when photographed from the shore of Possession Island and from fishing vessels was positive and significant (Mantel test *R* = 0.18, *p*‐value <.001). The social differentiation was strong among the 79 individuals (*S* = 1.08, SE = 0.008) and the estimated association indices were a useful representation of the true association indices (*r* = 0.70, SE = 0.005). With *H* = 74.02 and *S* ^2^ × *H* = 86.47, our data provided sufficient power to test the null hypothesis of individuals randomly associating with each other, and permutation tests supported the preferred/avoided association hypothesis (observed CV = 1.54, mean random CV = 1.38, *p*‐value <.005; Table 1).

**TABLE 1:** Permutation test statistics for non‐random associations among the killer whales of the Crozet Islands using 79 individuals between 2005 and 2022 considered as alive at the end of the study period.

Test statistic	Real value	Mean of permuted values	*p*‐value
CV of SRI	1.54	1.38	.005
SD of SRI	0.13	0.11	.000
SD of nonzero SRI	0.13	0.11	.000

*Note*: *p*‐values were calculated as the proportion of times that the test statistic of the observed network is smaller than a randomised network.

Abbreviations: CV, Coefficient of Variation; SD, Standard Deviation; SRI, Simple Ratio Index.

**FIGURE 1:**
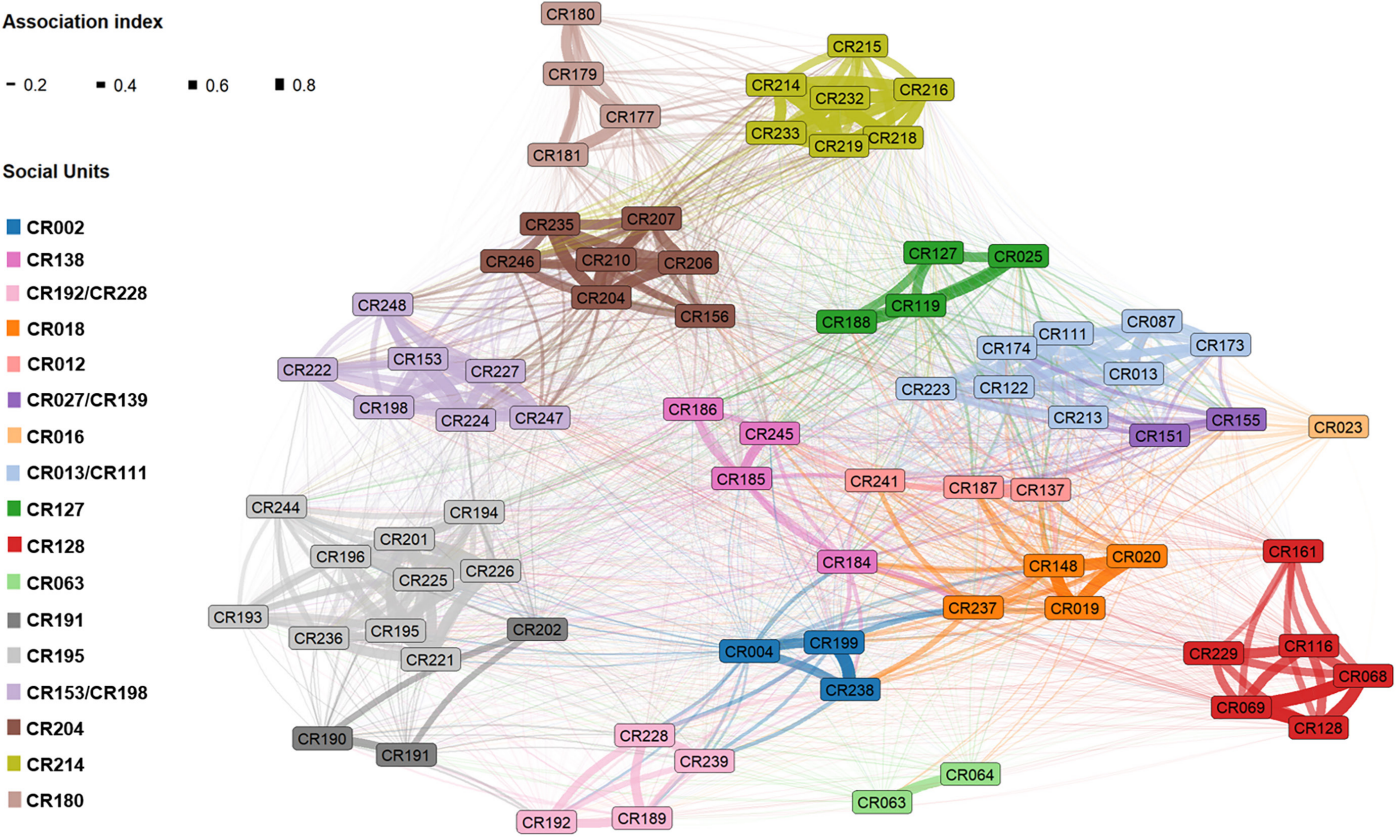
Network graph showing the associations among the 79 killer whales of the Crozet Islands used for the study between 2005 and 2022. Individuals are represented by nodes (coloured rectangles) and associations by edges (lines) between nodes. Colours represent social units and edges are weighted by the simple ratio index (SRI). The alpha‐numeric codes used to identify individuals and social units are from the photo‐identification catalogue of the population (Tixier, Gasco, et al., 2021). The graph was laid out using the ForceAtlas2 algorithm in R (Jacomy et al., 2014).

All individuals were connected to a single social network and 17 social units were detected within this network for the Crozet killer whale population (Figure 1). This social structuration from the Constant Potts Model (Leiden Algorithm) was significantly stable and well fitted with the optimal gamma resolution parameter (γ = 0.28; Appendix S2: Figure S1). The social units included between 1 and 10 individuals, with a mean size of 4.7 ± 2.5 SD individuals (*n* = 17 social units). The mean SRI within units was >0.5 for 10 units (*CR214*, *CR063*, *CR127*, *CR002*, *CR180*, *CR018*, *CR204*, *CR153/CR198*, *CR128*, *CR192/CR228*) and between 0.3 and 0.5 for 6 units (*CR013/CR111*, *CR012*, *CR027/CR139*, *CR138*, *CR191*, *CR195*). One unit (*CR016*) was made of a single individual considered as still alive at the end of the study period (Appendix S2: Figure S2).

### 3.2 Heterogeneity in the extent of depredation between social units

**FIGURE 2:**
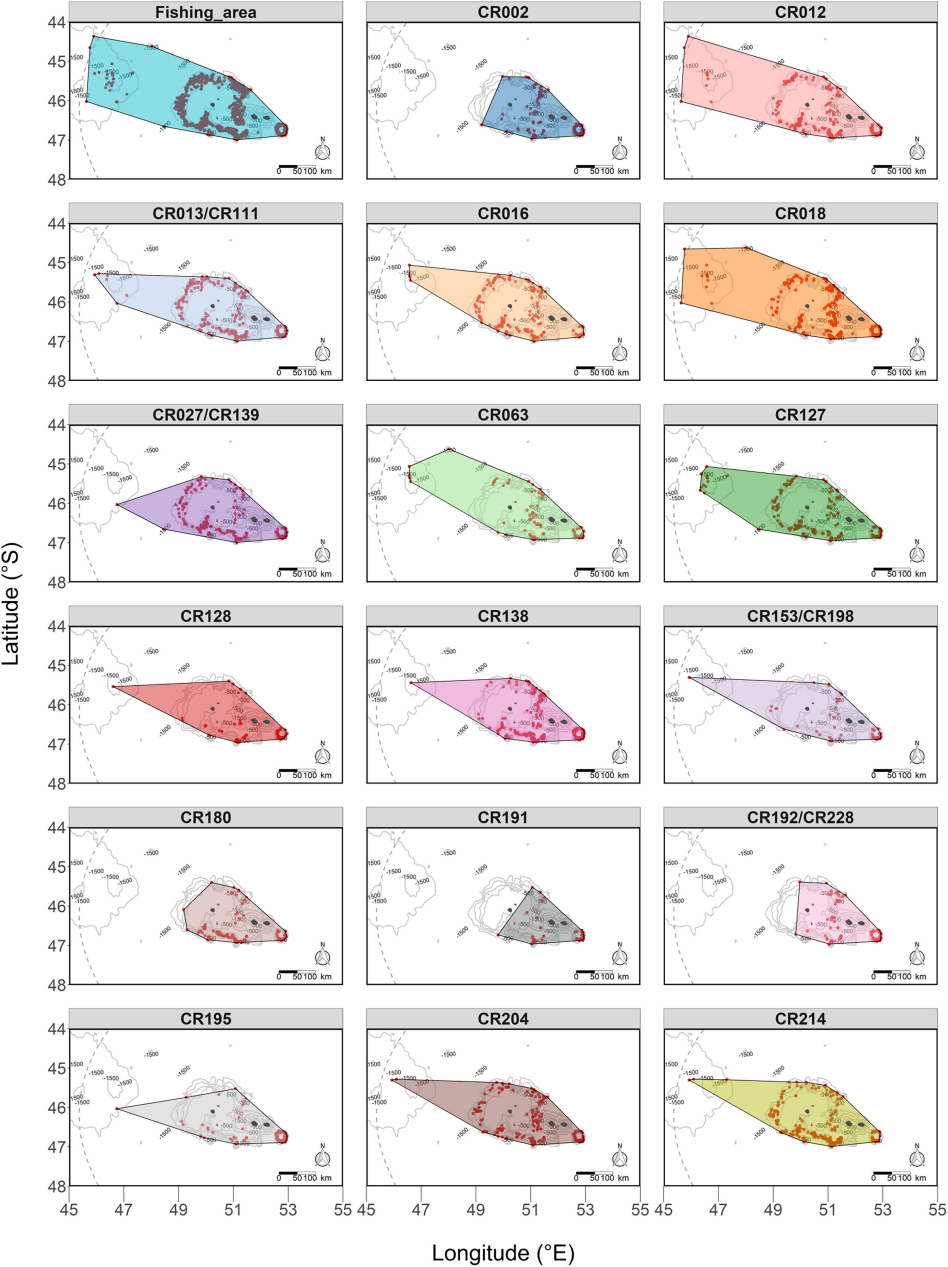
The spatial range of the depredation events during which killer whale social units were sighted (red points) estimated by minimum convex polygons (MCPs), around the Crozet Islands between 2005 and 2022.

**FIGURE 3:**
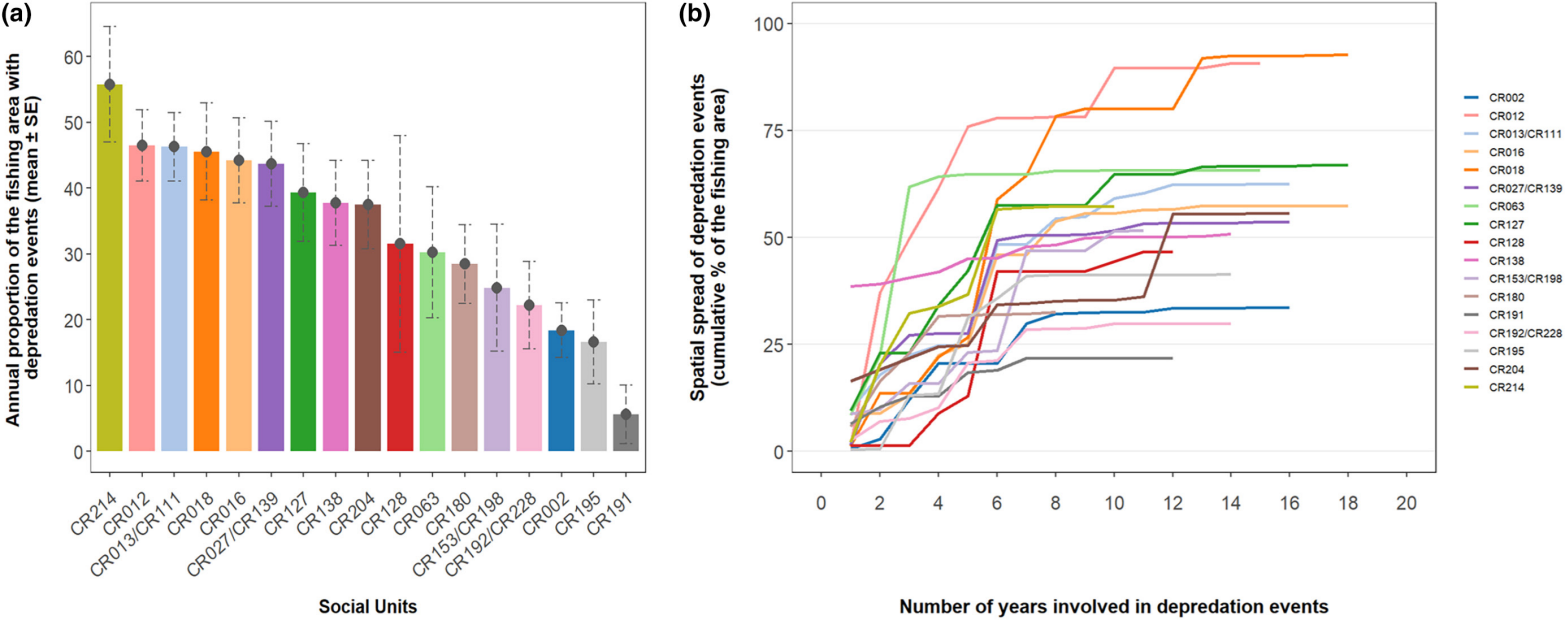
Proportion of the fishing area as estimated from minimum convex polygons (MCPs) in which social units were sighted during depredation events around the Crozet Islands between 2005 and 2022. (a) Annual mean proportion (% ± Standard Error (SE)) of the fishing area over which each social unit was sighted during depredation events; (b) cumulative proportion of the fishing area over which each social unit was sighted during depredation events over the years following the year social units were first sighted during a depredation event.

Between 2005 and 2022, the 17 social units were photographed during depredation events over varying spatial ranges. MCPs for units sighted over 17 years ranged from 33.3% of the fishing area (unit *CR002*) to 90.7% (unit *CR012*; Figure 2, Table 2). The annual mean of this proportion ranged from 18.4% ± 4.1 SE of the fishing area (unit *CR002*, *n* = 17 years) to 55.8% ± 8.7 SE (unit *CR012*, *n* = 17 years) (Figure 3a).

**TABLE 2:** Summary table of the number of years each of the 17 killer whale social units of the Crozet Islands was sighted during depredation events between 2005 and 2022, the total number of depredation events (sightings from fishing vessels) during which each unit was photographed, the proportion of these sightings out of all sightings during which any social unit was photographed from fishing vessels, the Minimum Convex Polygon (MCP) area in km^2^ and the proportion of the fishing area in which they were sighted during depredation events, the number of 0.1° × 0.1° spatial cells and the proportion of the fishing area in which they were sighted during depredation events, the depredation spatial range associated with core and representative area (km^2^) estimates using kernel utilisation density (UD) respectively at 50% and 95%.

Social unit	Number of years sighted	Total number of sightings	Proportion of all sightings (%)	MCP area (km^2^)	% of the fishing area (MCP) with sightings	Number of spatial cells with sightings	% of the fishing area (spatial grid) with sightings	Area (km^2^) – kernel UD (50%)	Area (km^2^) – kernel UD (95%)
CR002	17	189	12.8	32,614	33.6	56	24.9	8073	46,123
CR012	17	308	20.9	88,109	90.7	101	44.9	18,398	90,709
CR013/CR111	18	368	24.9	60,725	62.5	122	54.2	15,484	80,156
CR016	15	220	14.9	55,732	57.4	102	45.3	15,739	78,274
CR018	18	400	27.1	90,053	92.7	121	53.8	13,322	79,674
CR027/CR139	16	268	18.2	52,061	53.6	98	43.6	14,792	72,356
CR063	12	102	6.9	63,818	65.7	55	24.4	23,140	113,273
CR127	18	217	14.7	64,982	66.9	104	46.2	24,776	110,112
CR128	12	77	5.2	45,323	46.7	33	14.7	11,985	70,233
CR138	16	346	23.5	49,414	50.9	84	37.3	8751	54,111
CR153/CR198	13	86	5.8	50,115	51.6	36	16.0	13,585	85,293
CR180	10	157	10.6	31,543	32.5	53	23.6	8765	46,259
CR191	12	63	4.3	21,142	21.8	25	11.1	7868	42,251
CR192/CR228	16	145	9.8	28,927	29.8	42	18.7	7376	39,523
CR195	16	186	12.6	40,217	41.4	39	17.3	3212	40,802
CR204	17	238	16.1	53,992	55.6	101	44.9	18,123	78,294
CR214	10	329	22.3	55,669	57.3	110	48.9	11,973	72,268

**FIGURE 4:**
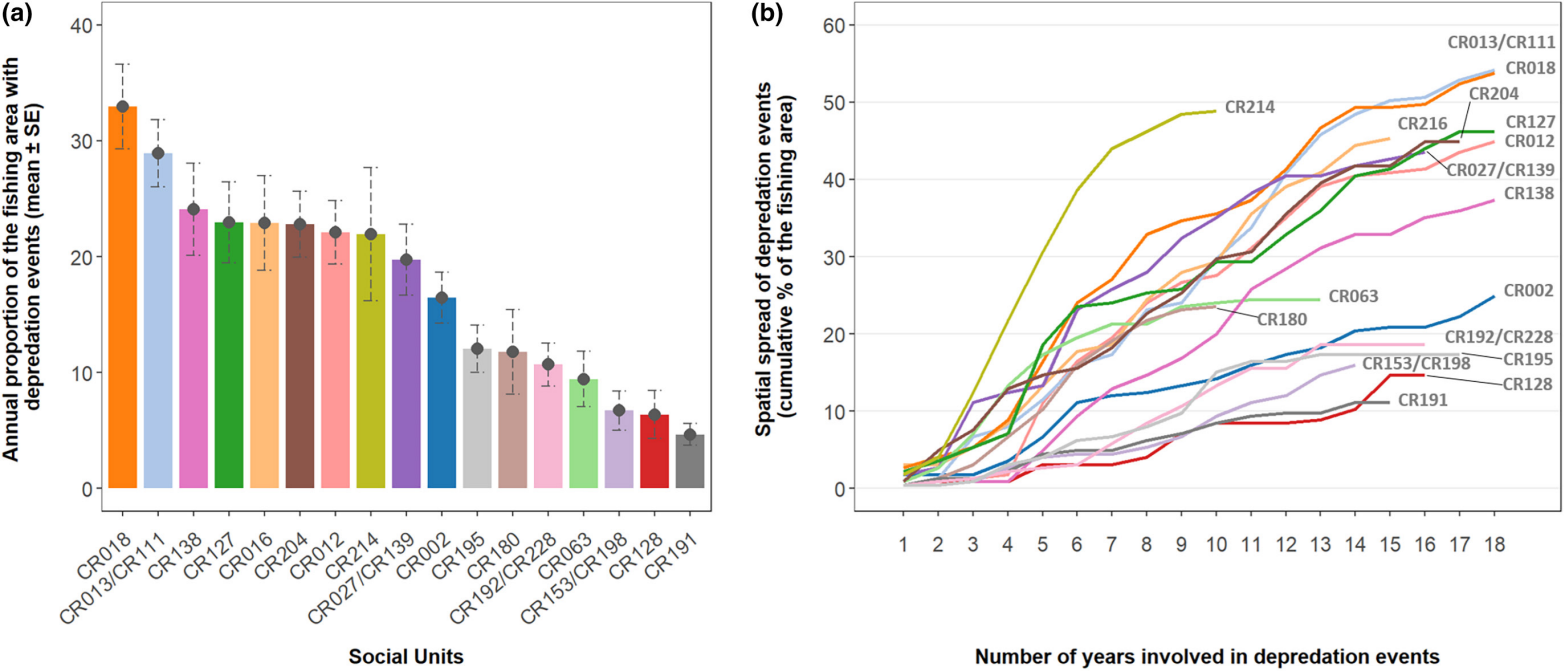
Proportion of the fishing area as estimated from the number of spatial cells in which social units were sighted during depredation events around the Crozet Islands between 2005 and 2022. Sightings were gridded in 0.1° × 0.1° spatial cells. (a) Annual mean proportion (% ± standard error (SE)) of the fishing area over which each social unit was sighted during depredation events; (b) cumulative proportion of the fishing area over which each social unit was sighted during depredation events over the years following the year social units were first sighted during a depredation event.

Using the number of spatial cells in which social units were sighted during depredation events, for the same number of years they were sighted (*n* = 16 years), unit *CR192/CR228* was sighted during depredation events over 10.7% ± 1.8 SE of the fishing area while unit *CR138* was sighted over 24.1% ± 4.0 SE of the fishing area (Figure 4a, Table 2, Appendix S2: Figure S3). The UD95 of social units ranged from 39,523 to 113,273 km^2^, and the UD50 of social units ranged from 3212 to 24,776 km^2^ (Table 2, Appendix S2: Figure S4). No correlation was found between the number of years social units sighted and the total area over which they were sighted for the MCP, UD95, UD50 (Spearman's rank correlation test: rho (MCP) = 0.47, *p*‐value = .06; rho (UD95) = 0.30, *p*‐value = .24; rho (UD50) = 0.30, *p*‐value = .25), and this correlation was slightly significant for the number of spatial cells (Spearman rank correlation test: rho (spatial cells) = 0.56, *p*‐value = .02; Table 2, Appendix S2: Figure S5).

From the first year they were sighted during a depredation event, social units expanded the spatial range of the depredation events during which they were sighted at varying rates (Figures 3b, 4b). From MCPs, while units like *CR191* were sighted during depredation events over <25% of the fishing area 12 years after their first sighting, others like units *CR012* and *CR018* had expanded this range to >75% of the fishing area over the same time period (Figure 3b). From the number of spatial cells, while units like *CR191* were sighted during depredation events over <20% of the fishing area 14 years after their first sighting, others like units *CR013/CR111* and *CR018* had expanded this range to >40% of the fishing area over the same time period (Figure 4b).

Over the study period, the proportion of depredation events during which social units were sighted varied from 4.3% of all sightings for unit *CR191* to 27.1% for unit *CR018* (Table 2). Such variation was also found across units that were sighted over the same number of years. For example, for units that were sighted over 16 years, the proportion of depredation events during which social units were sighted varied from 9.8% of all sightings for unit *CR192/CR228* to 23.5% for *CR138* (Table 2). Out of the 17 social units involved in depredation events, 4 units (*CR018*, *CR013/CR111*, *CR138*, *CR214*) were, together, sighted during 71% of all depredation events (Table 2).

The null GLMM fitted to the occurrence of social units during depredation events showed no overdispersion (dispersion ratio = 1, *p*‐value = .497). The best‐fitted model included all fixed terms (Model 4 in Table 3, AICc = 14,999.30, Conditional *R* ^2^ = 13.64, *X* ^2^ = 699, Pr (>*X* ^2^) <.001) and was validated by testing for multicollinearity (VIF <3 for all terms), zero‐inflation (ratioObsSim = 1, *p*‐value = .616), autocorrelation (Durbin Watson test: DW = 1.94, *p*‐value = .275), linearity and extreme values (KS test = 0.73, Outlier test = 0.64). The addition of a nested structure within a random effect to address pseudoreplication did not improve the model fit (Model 5 in Table 3, AICc = 15,003.31, Conditional *R* ^2^ = 13.64, *X* ^2^ = 0, Pr (>*X* ^2^) = 1). From this, we selected Model 4 as the best‐fitted model to adhere to the principle of parsimony and ensure a balance between model complexity and interpretability.

**TABLE 3:** Comparison of model outputs for the five GLMMs fitted to the occurrence of killer whale depredation (Depred) with the photographic effort (CP), sighting (SC) and social unit are random terms, with the social unit nested within sighting.

Model parameters	Model rank	*K*	LL	AICc	wAICc	*R* ^2^ m	*R* ^2^ c	*X* ^2^	df	Pr (>*X* ^2^)
Null: Depred ~1 + (1|CP)	1	2	−7848.32	15700.65	0.00	0.00	1.82	/	/	/
Depred ~ year + (1|CP)	2	19	−7823.80	15685.63	0.00	0.65	2.60	49.06	17	<.001
Depred ~ year + month + (1|CP)	3	30	−7803.06	15666.21	0.00	1.20	3.33	41.47	11	<.001
Depred ~ year + month + unit + (1|CP)	4	46	−7453.54	14999.30	0.88	11.60	13.64	699.03	16	<.001
Depred ~ year + month + unit + (1|CP) + (1|SC/unit)	5	48	−7453.54	15003.31	0.12	11.60	13.64	0.00	2	1

*Note*: The year, the month and the ID of the killer whale social unit sighted during depredation events (unit) as fixed terms. The outputs include AICc, Akaike information criterion; DF, degree of freedom; *K*, the number of parameters; LL, Likelihood; Pr, probability; *R* ^2^c, conditional *R* ^2^; *R* ^2^m, marginal *R* ^2^; wAICc, AIC weight; *X* ^2^, Chi‐square.

**FIGURE 5:**
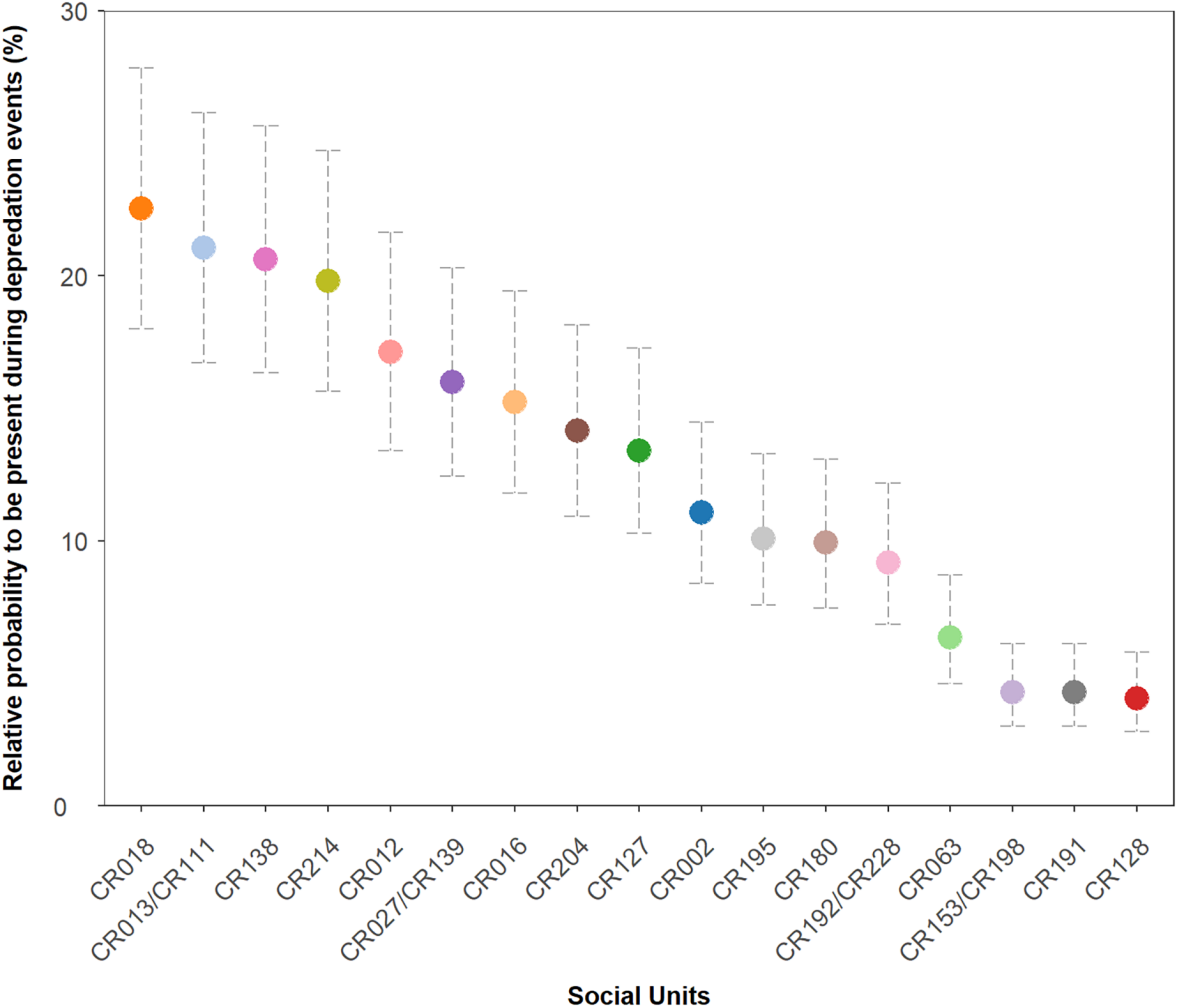
The relative probability of each of the 17 killer whale social units of the Crozet Islands to be present during depredation events between 2005 and 2022, as estimated by a GLMM fitted to the occurrence of killer whale social units during depredation events. Error bars are 95% confidence intervals.

The relative probability of social units to be present during depredation events, as estimated from the final model, significantly varied between social units (post hoc mean comparisons across all social unit pair combinations, *p*‐value <.05; Appendix S2: Figure S6, Table S1). It ranged from 4.0% [95% CI 2.8–5.8] for units *CR128*, *CR191* and *CR153/CR198* to 22.5% [95% CI 18.0–27.8] for unit *CR018* (Figure 5).

## 4 DISCUSSION

In this study, we highlight large behavioural heterogeneity in the way killer whale social units within a population may respond to new feeding opportunities from fisheries. We found that social units of the Crozet killer whale population, which we identified as groups of 1–10 individuals with long‐lasting associations through a social network analysis, consistently with previous studies (Guinet, 1991, 1992; Tixier, Gasco, et al., 2021), were involved in depredation events at varying rates and over different spatial ranges (independently from the number of years units were sighted). Two patterns emerged from our findings. While all units were consistently sighted during at least one depredation event per year, some were more frequently present during these events and over a greater proportion of the fishing area than others, which were only sporadically present and were so in the same small areas. Intra‐population variation in the way individuals use fisheries to feed has been observed in seabirds (Patrick et al., 2015; Votier et al., 2010), seals (Cronin et al., 2016; Graham et al., 2011; Königson et al., 2013) and odontocetes (Anderson et al., 2020; Baird et al., 2015, 2019; Genov et al., 2019). Specifically, heterogeneity in the frequency at which and/or the spatial range over which individuals depredated on fisheries catches, i.e., the two indices we used, was shown within grey seal *Halichoerus grypus* populations depredating on static nets in the Irish and Celtic seas (Cronin et al., 2016) and bottlenose dolphins *Tursiops truncatus* depredating on trawl nets in the northern Adriatic Sea (Genov et al., 2019).

Intra‐population heterogeneity in the depredation behaviour of killer whale social units can be driven by multiple factors, acting alone or together. Firstly, existing variation in the natural distribution of individuals may lead to varying degrees of overlap with fishing activities and, therefore, to differences in the probability of individuals to be present during depredation events. This was, for instance, shown across social clusters of false killer whales *Pseudorca crassidens* within the population depredating on longline catches around Hawaii (Baird et al., 2019). For killer whales at Crozet, this assumption is supported by (i) variation in the spatial distribution of depredation events between units (this study) and (ii) evidence that social units use the area differently from data collected in inshore waters, with for instance, elephant seal *Mirounga leonina* colonies of Possession Island being used as foraging grounds by only a subset of the units of the population (Guinet et al., 2015; Tixier, Gasco, et al., 2021). Secondly, it can be driven by existing variation in the level of specialisation of individuals to their natural prey items. On the one hand, and in line with the previous assumption, some social units may be more specialised into toothfish and, therefore, more likely to overlap and compete with fishing vessels for that resource than others. On the other hand, some social units may be more likely to depredate fish caught on fishing gear as being more generalist in their feeding preferences, and thus more opportunistic in their foraging behaviours and more likely to switch to depredation than others. Such specialisation gradient, which is commonly observed within populations of generalist species as a mechanism to lower intraspecific competition (Araújo et al., 2011; Bolnick et al., 2011), has been demonstrated in other killer whale populations (Jourdain et al., 2020; Samarra et al., 2017) and is likely to occur across killer whale units at Crozet population given the broad range of prey consumed as a population (Tixier et al., 2019). Thirdly, heterogeneity in the extent to which social units are involved in depredation events may be explained by the social affinity between these units. As most behaviours of killer whales are socially learnt, it is possible social units preferentially associating with each other homogenise their foraging strategies, including depredation, through horizontal (intra‐generational) transmission. Such homogenisation across strongly associated individuals was for instance, shown in the crop‐raiding behaviour of elephants *Loxodonta africana* (Chiyo et al., 2012). Lastly, the behaviour of killer whale groups, as a whole, may be influenced by the intrinsic characteristics of their leader or decision‐maker (likely matriarchs; Brent et al., 2015) such as their experience but also their personality (also referred to as “behavioural syndrome” and defined as suites of correlated behaviours expressed either within a given behavioural context or across different contexts (Sih et al., 2004)), as shown in other highly social species (McComb et al., 2011; Toscano et al., 2016; Troxell‐Smith & Mella, 2017).

Our findings have implications for both the conservation of Crozet killer whales and their ecosystem, as well as for the mitigation of the conflict associated with the depredation of fishery catches. For killer whales, social units present during depredation events more frequently and over a greater range are more likely to encounter fishing vessels operating illegally in the region (i.e., out and inside the Crozet EEZ (Weimerskirch et al., 2020)) and, therefore, to be exposed to killing practices potentially used from these vessels to reduce depredation (Tixier, Gasco, et al., 2021). For these social units, the intake of depredated toothfish to their diet may also be greater, and this may lead to stronger effects of food provisioning on their demographic rates (Tixier et al., 2017) and on their ecological role in the ecosystem (e.g., alteration of their predatory pressures on other prey; Clavareau et al., 2020, 2023). The 179 tons of toothfish removed annually by killer whales from longlines at Crozet were shown to contribute 8.8% of the annual energetic requirements of the whole killer whale population (Faure et al., 2021; Tixier et al., 2020), but from our findings, this contribution is likely to greatly vary across social units. In particular, this contribution and its subsequent demographic and ecological effects are likely maximum for the 4 social units out of 17 that we found involved in more than 70% of the depredation events. Heterogeneity in the extent to which killer whale social units are involved in depredation events may also influence the effectiveness of avoidance strategies implemented by fishers to mitigate depredation. For example, moving on to another area after being subject to killer whale depredation is a strategy that may not work with social units that we identified here as frequently depredating over large areas because these units may be more inclined to actively search and/or follow vessels over great distances.

In conclusion, from the case study of killer whales depredating toothfish on longlines around the Crozet Islands, we showed that killer whale social units can respond differently to human‐induced changes in prey availability in their environment. This intra‐population behavioural heterogeneity translates into large variation in the extent to which these units interact with fishing vessels to feed on fish caught on the fishing gear. Although the drivers of such heterogeneity are still unclear and should be investigated in the future, our findings suggest that fisheries have varying levels of impact on the life‐history traits of individuals within the population. This has major implications on conservation and evolutionary trajectories of killer whale populations, at Crozet where killer whale numbers have drastically declined over the past 30 years, but also for the many killer whale populations that have been reported depredating on fisheries catches worldwide.

## AUTHOR CONTRIBUTIONS

**Erwan Auguin:** Conceptualization (equal); formal analysis (equal); investigation (equal); methodology (equal); validation (equal); visualization (equal); writing – original draft (lead); writing – review and editing (equal). **Christophe Guinet:** Conceptualization (equal); data curation (equal); investigation (equal); supervision (equal); validation (equal); visualization (equal); writing – review and editing (equal). **Johann Mourier:** Formal analysis (equal); methodology (equal); validation (equal); visualization (equal); writing – review and editing (equal). **Eric Clua:** Funding acquisition (equal); project administration (lead); validation (equal); visualization (equal); writing – review and editing (equal). **Nicolas Gasco:** Data curation (equal); resources (equal); writing – review and editing (equal). **Paul Tixier:** Conceptualization (equal); data curation (equal); formal analysis (equal); funding acquisition (equal); investigation (equal); methodology (equal); supervision (equal); validation (equal); visualization (equal); writing – review and editing (equal).

## FUNDING INFORMATION

The study was funded by Agence Nationale de la Recherche (Project EthoPredator #21‐CE03‐0004).

## CONFLICT OF INTEREST STATEMENT

We declare we have no competing interests.

## Supporting information


Appendices S1–S3.

## Data Availability

The data used for the study were provided as two spreadsheets – online resource: https://doi.org/10.6084/m9.figshare.24442669.v1.
