# Supplementary material for: Behavioural heterogeneity across killer whale social units in their response to feeding opportunities from fisheries
Source: Ecol Evol. 2024 May 23;14(5):e11448. doi: 10.1002/ece3.11448 (PMC11116761; doi:10.1002/ece3.11448)
Supplement: Supplementary file 1 — Appendices S1–S3. [file ECE3-14-e11448-s001.docx]

*The following supplements accompany the article*

**Behavioural heterogeneity across killer whale social units in their response to feeding opportunities from fisheries**

**Erwan Auguin*. Christophe Guinet. Johann Mourier. Eric Clua. Nicolas Gasco. Paul Tixier**

*Corresponding author: [erwan.auguin@ird.fr](mailto:erwan.auguin@ird.fr)

*Ecology and Evolution*

**This document includes:**

Appendix S1_ Supporting text for testing social relationships

Appendix S2_ Figures S1 to S6 and Table S1

Appendix S3_ References

Appendix S1

**Supporting text** **for testing social relationships**

We calculated the social differentiation (S) as the variability of the “true” association indices estimated using maximum likelihood approximation (Whitehead, 2008a, 2008b). S values close to “0” indicate homogeneous relationships within the population, while values near or greater than “1” indicate a highly differentiated society (Whitehead, 2008a). To determine the accuracy of association indices and their power in testing for social relationships, we calculated the correlation coefficient r as the ratio between S and the observed association indices (r= S/Coefficient of Variation (CV). Sufficient statistical power to test for preferred and avoided association is generally accepted when S² × H >5 (where H is the mean number of identifications per individual) (Table 1 in the main text) (Whitehead, 2008b).

Appendix S2


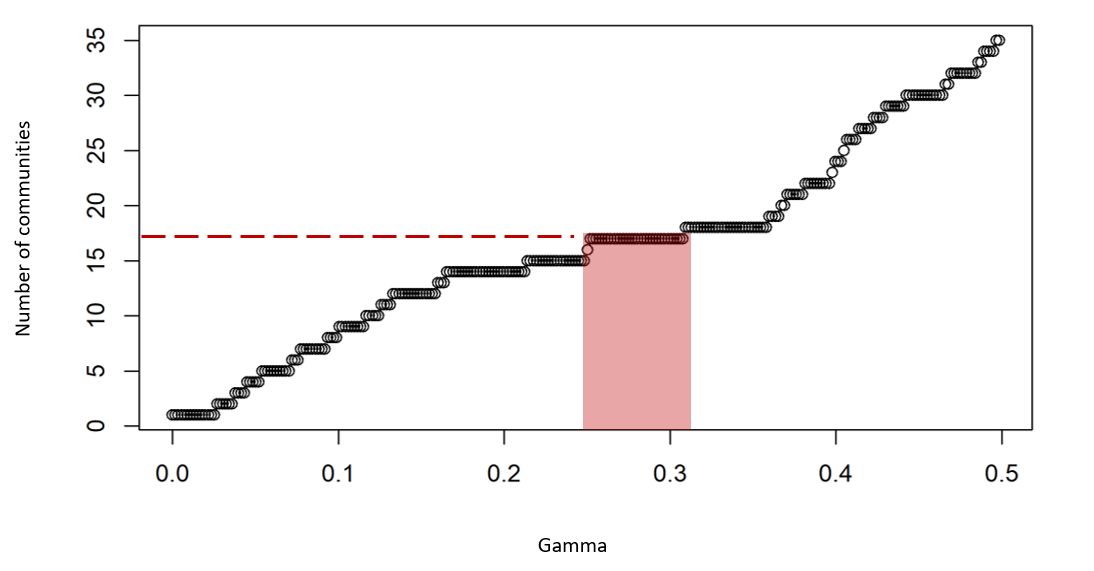


**Figure S1.** Determination of the number of social units (“communities”) as detected in the Crozet killer whale population between 2005 and 2022 from the Leiden algorithm by Constant Potts Model (CPM) with changing quality factor (“Gamma” resolution). The red shade represents the optimal “Gamma” resolution parameters for robust detection of social units, characterised by a very stable plateau over a large “Gamma” variation scale (Güldal, 2021). In our case, this corresponded to 17 killer whale social units.


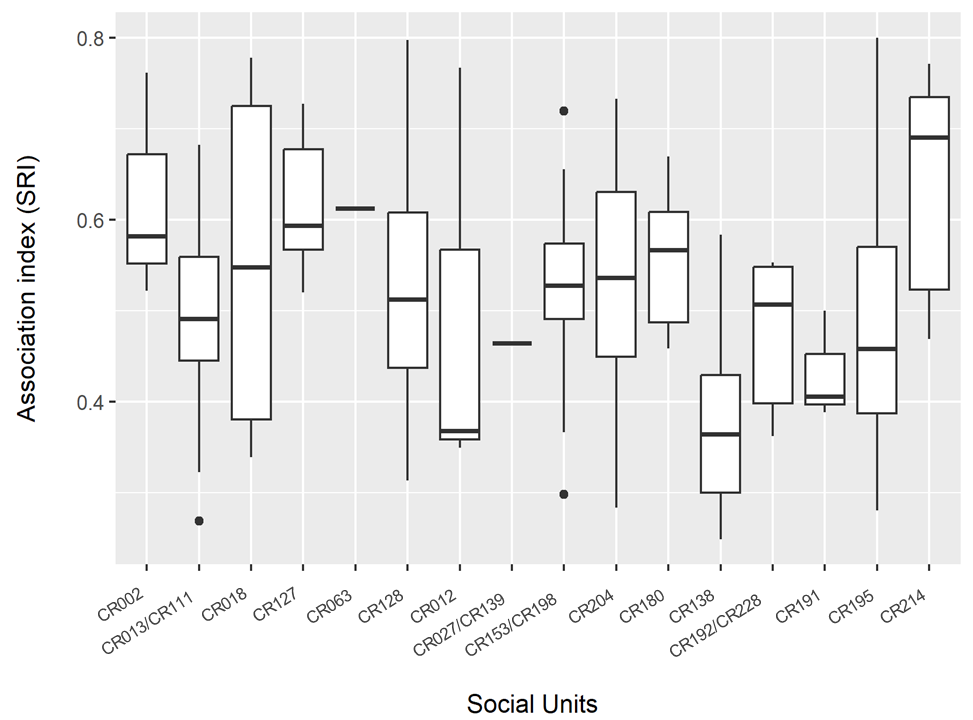


**Figure S2.** Boxplots of association index (Simple Ratio Index - SRI) values between individuals within the social units of the Crozet killer whale population between 2005 and 2022. These were calculated for 16 of the 17 social units because one unit (*CR016*) included only one individual.


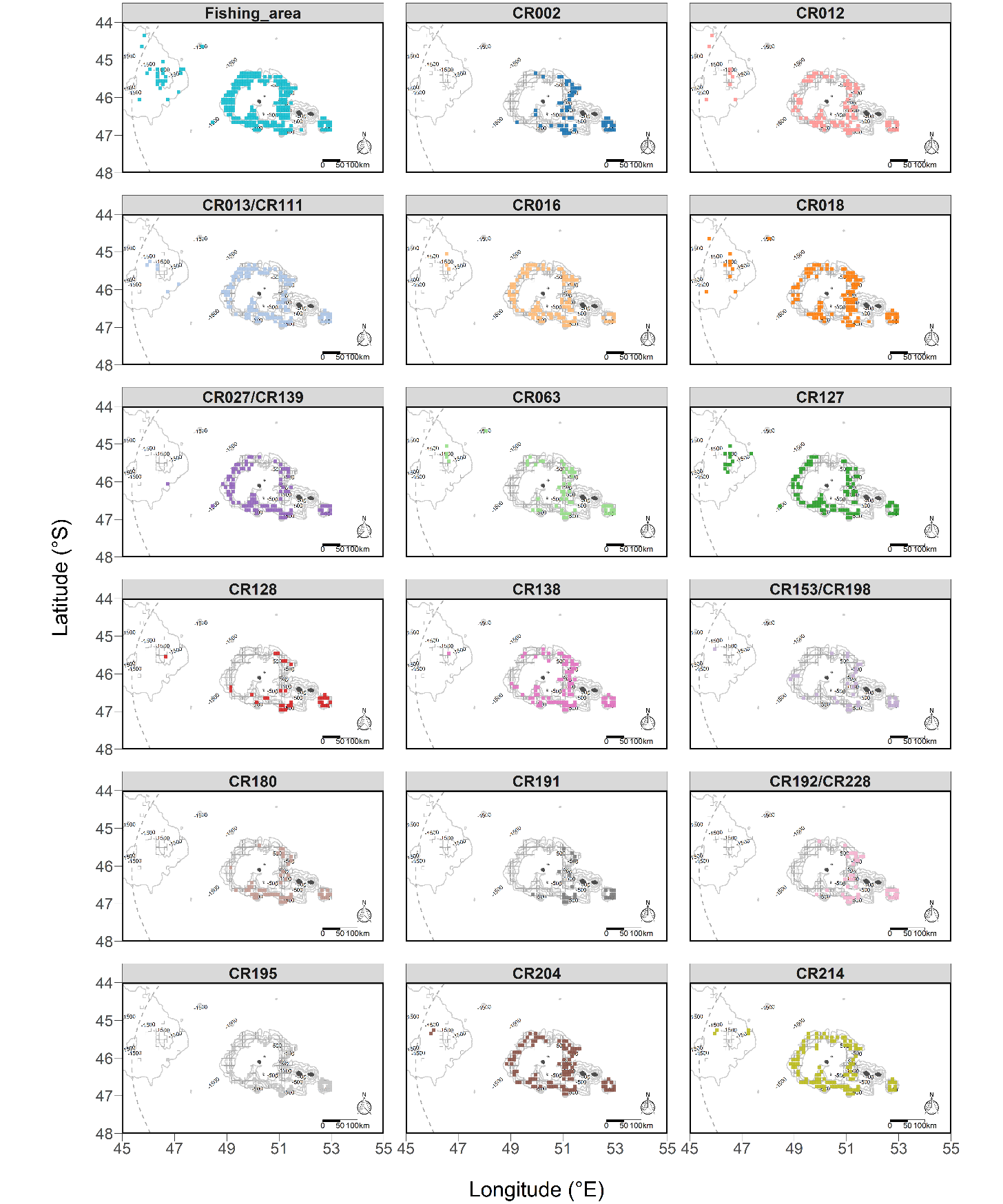


**Figure S3.** Spatial distribution of the depredation events (sightings from fishing vessels) during which killer whale social units were sighted around the Crozet Islands between 2005 and 2022. Sightings were gridded in 0.1° × 0.1° spatial cells, with turquoise blue: the sightings for all social units (referred to as the “fishing area” in the main text), and others colours: the sightings for each social unit.


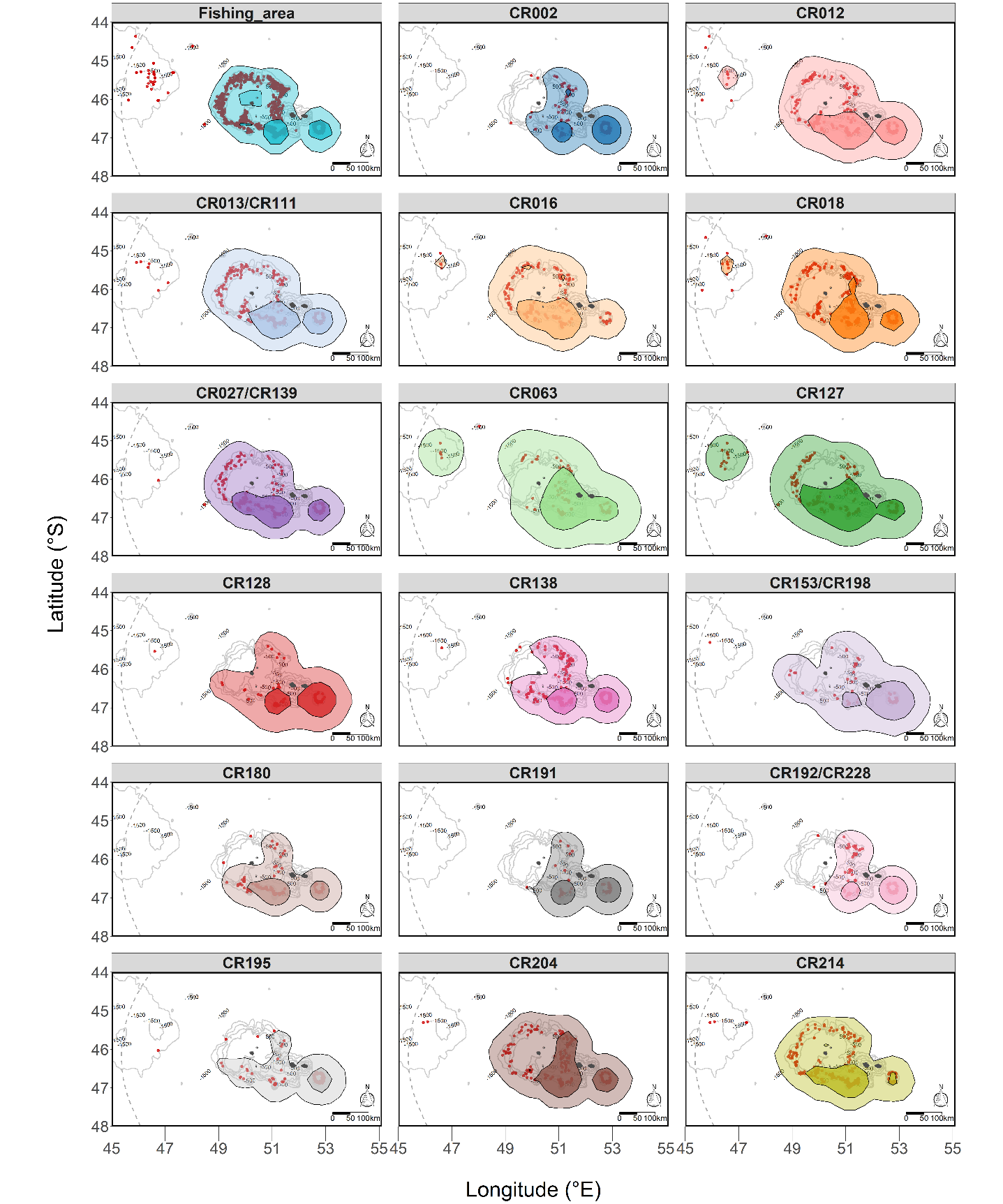


**Figure S4.** Spatial range of the depredation events (sightings from fishing vessels - red points) during which killer whale social units were present around the Crozet Islands between 2005 and 2022, as estimated by Kernel Density Estimation (KDE) to measure the Utilization Distribution (UD) in km2 at 50% (UD50) and 95% (UD95) (Worton, 1989). UD95 (light colour) and UD50 (dark colour) were defined as the smallest area containing 95% and 50% of the UD, respectively.


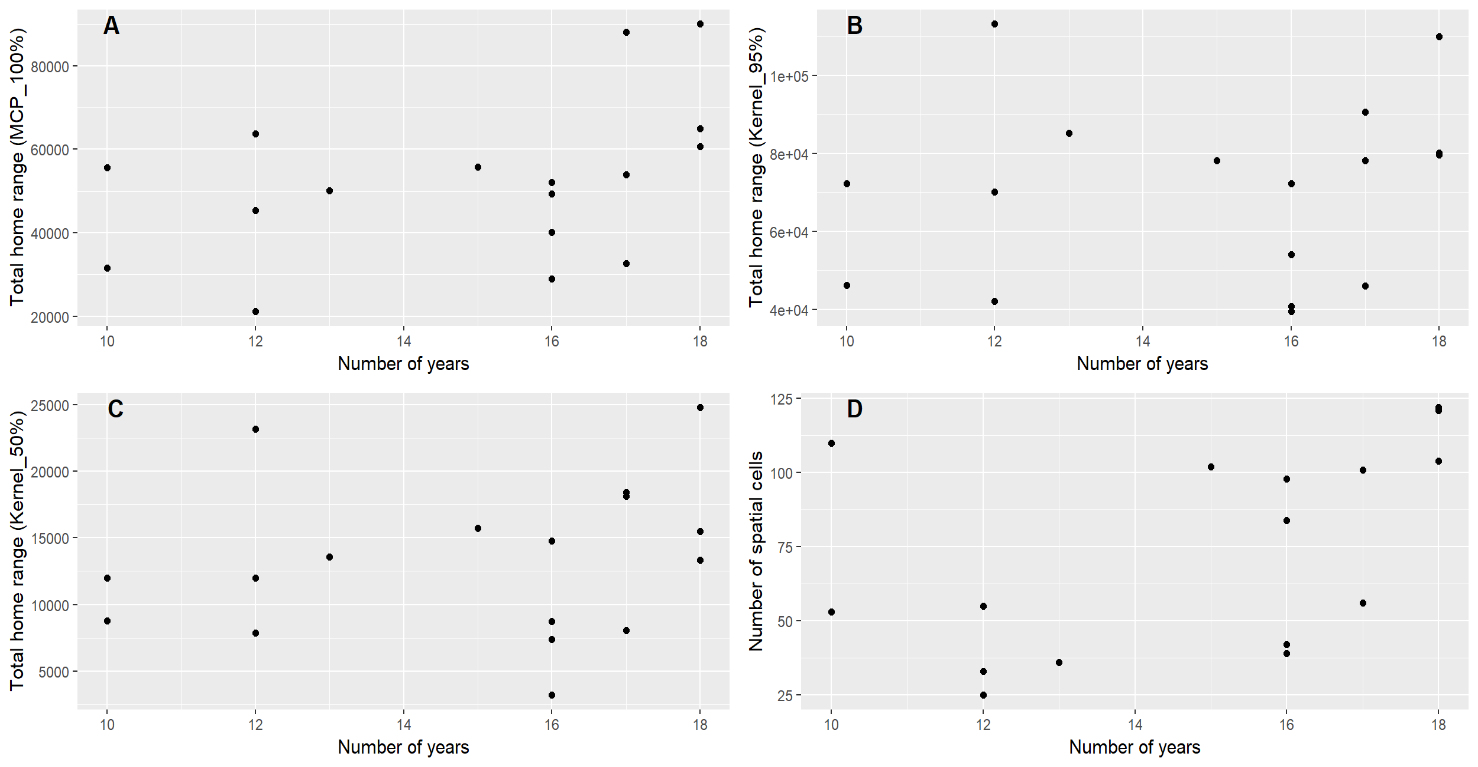

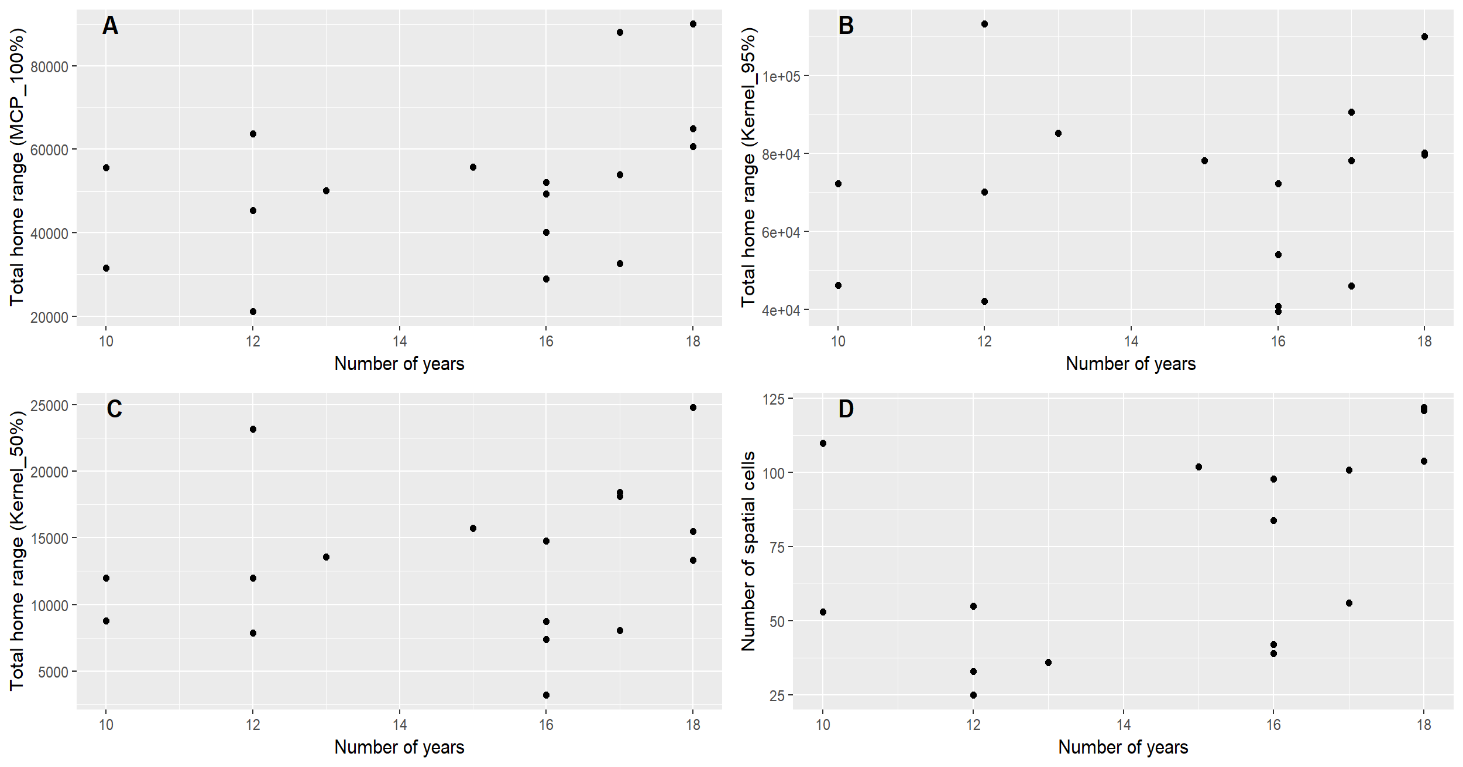

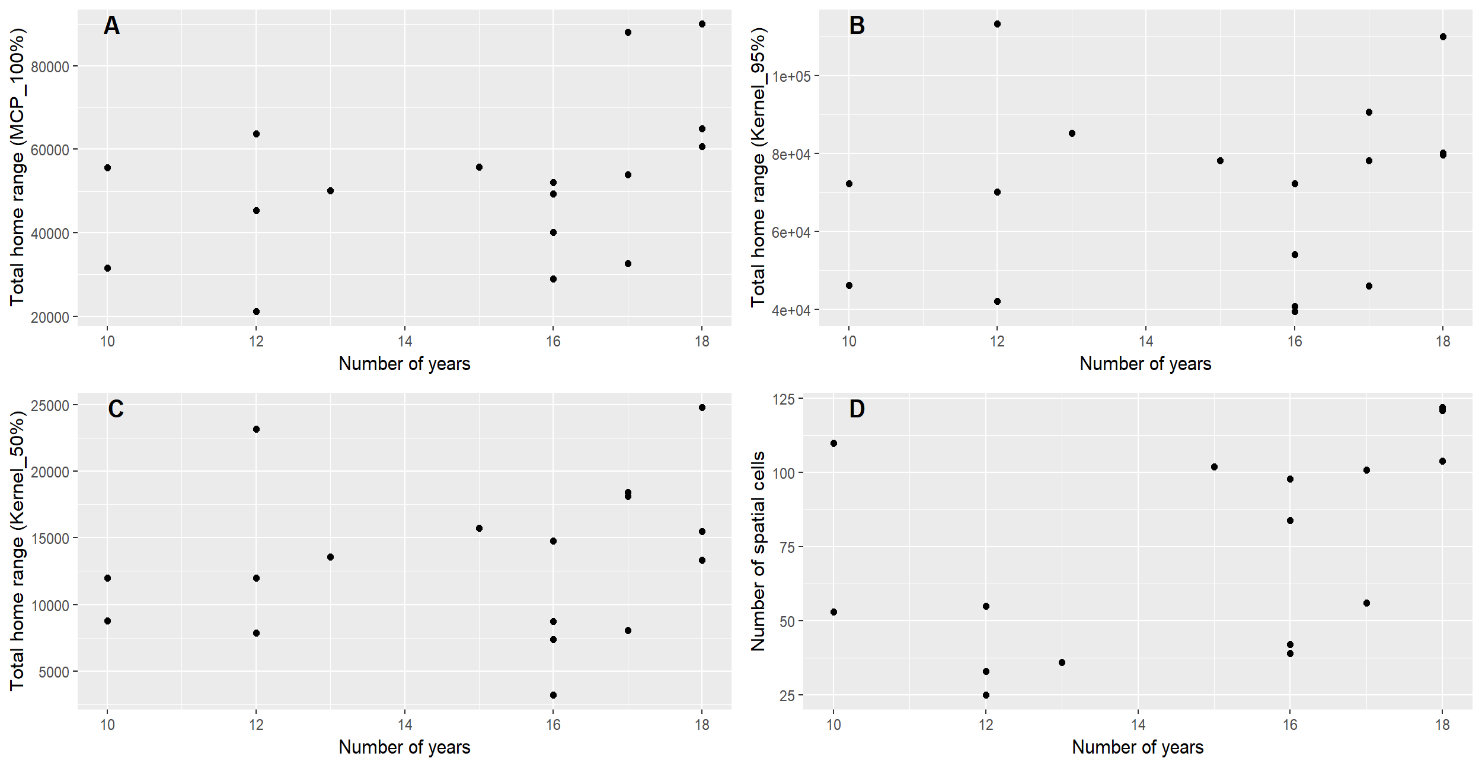

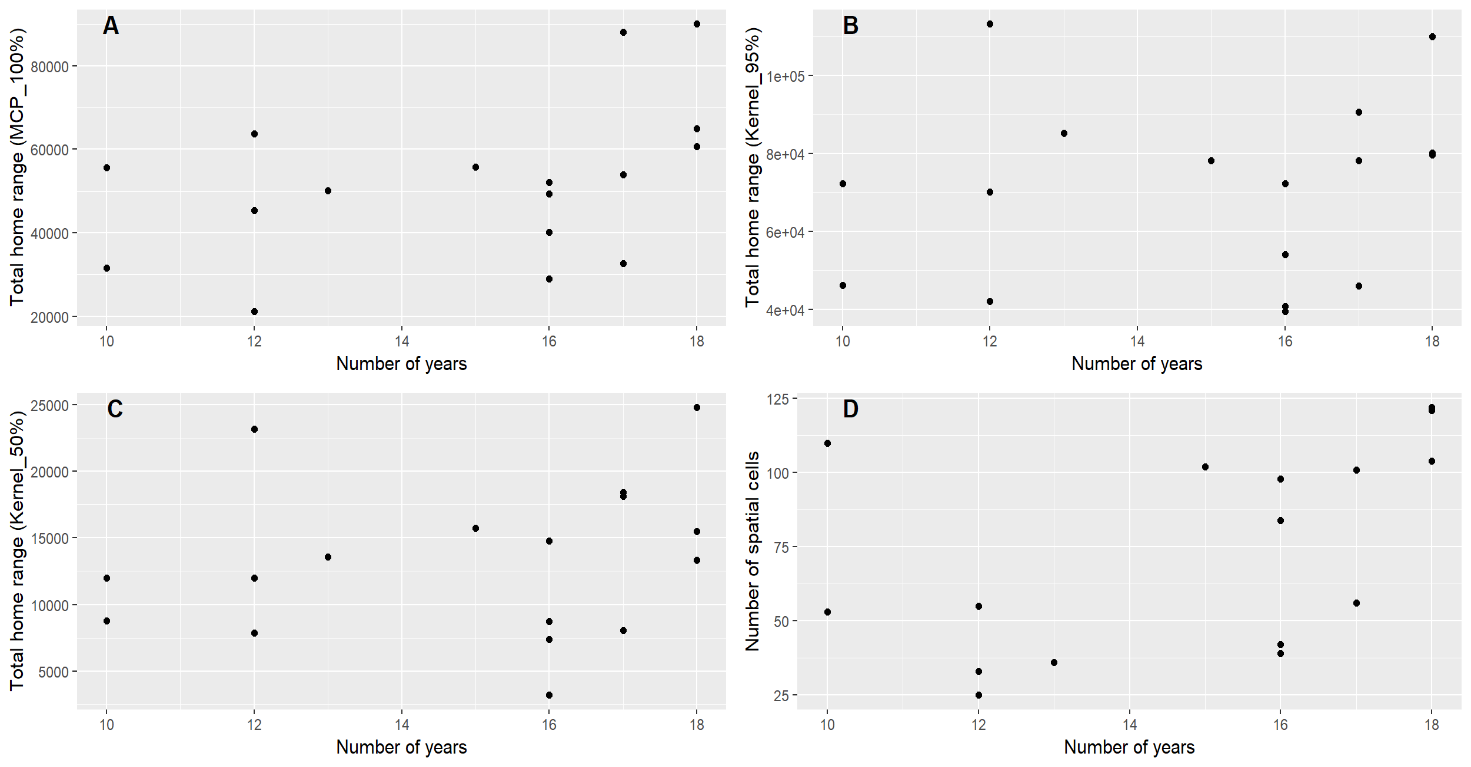


**Figure S5.** Scatter plots of the number of years killer whale social units were sighted during depredation events around the Crozet Islands between 2005 – 2022 as a function of the total area (km²) over which they were sighted for the Minimum Convex Polygon (MCP, Plot A), Utilisation Distribution at 95% (UD95, Plot B) and 50% (UD50, Plot C) and number of spatial cells (Plot D).


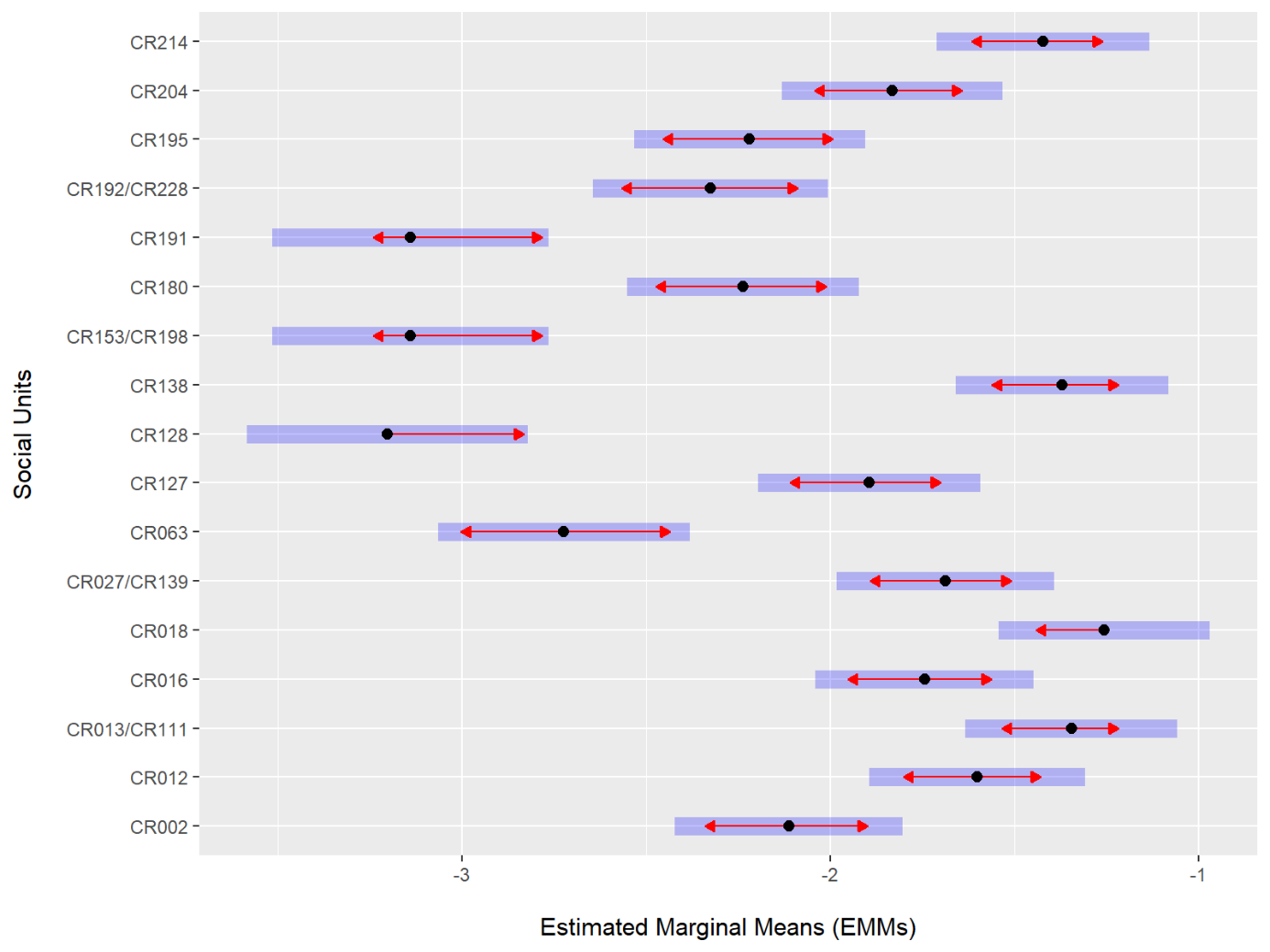


**Figure S6.** Estimated Marginal Means (EMMs) calculated from a categorial predictor (“Social Units”) within a Generalised Linear Mixed Model (GLMM) fitted to the occurrence of killer whale social units during depredation events at Crozet between 2005 and 2022 using a binomial distribution and a logit link function. The blue bars are confidence intervals for the EMMs. Red arrows indicate whether EMMs are significantly different between social units (arrows not overlapping) or not (arrows overlapping). EMMs were averaged over the others categorial predictors: “year”, “season” from final model (Table 3 in the main text) and based on the adjustment setting (“Holm”) and the value of alpha (0.05).

**Table S1.** Pairwise comparisons of Estimated Marginal Means (EMMs) across social units from the Generalised Linear Mixed Model (GLMM) fitted to the occurrence of killer whale social units during depredation events at Crozet between 2005 and 2022 using a binomial distribution and a logit link function. Model outputs included the *Estimate*: estimate of the EMMs differences for every combination of social units, *SE*: Standard Error of the *Estimate*, *Z-ratio*: the *Estimate* divided by its Standard Error with a comparison against the limiting normal distribution (an infinite number of degrees of freedom), *p-value* <0.05 if the null hypothesis (no significant difference between social units in their probability of being present during depredation events) is rejected. The table only presents the model outputs from combinations of social units with a significant difference in their probability of being present during depredation events.

| *Social units* | *Estimate* | *SE* | *Z-ratio* | *p-value* |
| --- | --- | --- | --- | --- |
| CR002 - CR012 | -0,511 | 0,120 | -4,262 | 0,001 |
| CR002 - (CR013/CR111) | -0,768 | 0,116 | -6,597 | 0,000 |
| CR002 - CR016 | -0,369 | 0,122 | -3,015 | 0,103 |
| CR002 - CR018 | -0,857 | 0,115 | -7,428 | 0,000 |
| CR002 - (CR027/CR139) | -0,426 | 0,121 | -3,512 | 0,022 |
| CR002 - CR063 | 0,610 | 0,150 | 4,076 | 0,003 |
| CR002 - CR127 | -0,218 | 0,125 | -1,744 | 1,000 |
| CR002 - CR128 | 1,089 | 0,173 | 6,307 | 0,000 |
| CR002 - CR138 | -0,742 | 0,117 | -6,361 | 0,000 |
| CR002 - (CR153/CR198) | 1,027 | 0,169 | 6,067 | 0,000 |
| CR002 - CR180 | 0,124 | 0,133 | 0,933 | 1,000 |
| CR002 - CR191 | 1,027 | 0,169 | 6,067 | 0,000 |
| CR002 - (CR192/CR228) | 0,211 | 0,136 | 1,555 | 1,000 |
| CR002 - CR195 | 0,106 | 0,133 | 0,797 | 1,000 |
| CR002 - CR204 | -0,282 | 0,124 | -2,274 | 0,689 |
| CR002 - CR214 | -0,690 | 0,117 | -5,880 | 0,000 |
| CR012 - (CR013/CR111) | -0,256 | 0,105 | -2,448 | 0,449 |
| CR012 - CR016 | 0,142 | 0,111 | 1,278 | 1,000 |
| CR012 - CR018 | -0,345 | 0,104 | -3,334 | 0,040 |
| CR012 - (CR027/CR139) | 0,085 | 0,110 | 0,771 | 1,000 |
| CR012 - CR063 | 1,122 | 0,141 | 7,958 | 0,000 |
| CR012 - CR127 | 0,293 | 0,115 | 2,556 | 0,371 |
| CR012 - CR128 | 1,600 | 0,165 | 9,692 | 0,000 |
| CR012 - CR138 | -0,231 | 0,105 | -2,198 | 0,755 |
| CR012 - (CR153/CR198) | 1,538 | 0,162 | 9,523 | 0,000 |
| CR012 - CR180 | 0,636 | 0,123 | 5,148 | 0,000 |
| CR012 - CR191 | 1,538 | 0,162 | 9,523 | 0,000 |
| CR012 - (CR192/CR228) | 0,723 | 0,126 | 5,728 | 0,000 |
| CR012 - CR195 | 0,617 | 0,123 | 5,021 | 0,000 |
| CR012 - CR204 | 0,229 | 0,113 | 2,027 | 1,000 |
| CR012 - CR214 | -0,179 | 0,106 | -1,689 | 1,000 |
| (CR013/CR111) - CR016 | 0,399 | 0,107 | 3,710 | 0,011 |
| (CR013/CR111) - CR018 | -0,089 | 0,099 | -0,895 | 1,000 |
| (CR013/CR111) - (CR027/CR139) | 0,342 | 0,106 | 3,211 | 0,058 |
| (CR013/CR111) - CR063 | 1,378 | 0,138 | 9,993 | 0,000 |
| (CR013/CR111) - CR127 | 0,549 | 0,111 | 4,958 | 0,000 |
| (CR013/CR111) - CR128 | 1,857 | 0,163 | 11,423 | 0,000 |
| (CR013/CR111) - CR138 | 0,026 | 0,101 | 0,253 | 1,000 |
| (CR013/CR111) - (CR153/CR198) | 1,795 | 0,159 | 11,294 | 0,000 |
| (CR013/CR111) - CR180 | 0,892 | 0,120 | 7,435 | 0,000 |
| (CR013/CR111) - CR191 | 1,795 | 0,159 | 11,294 | 0,000 |
| (CR013/CR111) - (CR192/CR228) | 0,979 | 0,123 | 7,976 | 0,000 |
| (CR013/CR111) - CR195 | 0,874 | 0,119 | 7,315 | 0,000 |
| (CR013/CR111) - CR204 | 0,486 | 0,109 | 4,443 | 0,001 |
| (CR013/CR111) - CR214 | 0,078 | 0,102 | 0,763 | 1,000 |
| CR016 - CR018 | -0,488 | 0,106 | -4,586 | 0,000 |
| CR016 - (CR027/CR139) | -0,057 | 0,113 | -0,508 | 1,000 |
| CR016 - CR063 | 0,979 | 0,143 | 6,849 | 0,000 |
| CR016 - CR127 | 0,150 | 0,117 | 1,285 | 1,000 |
| CR016 - CR128 | 1,458 | 0,167 | 8,737 | 0,000 |
| CR016 - CR138 | -0,373 | 0,108 | -3,462 | 0,026 |
| CR016 - (CR153/CR198) | 1,396 | 0,163 | 8,547 | 0,000 |
| CR016 - CR180 | 0,493 | 0,126 | 3,921 | 0,005 |
| CR016 - CR191 | 1,396 | 0,163 | 8,547 | 0,000 |
| CR016 - (CR192/CR228) | 0,580 | 0,128 | 4,518 | 0,000 |
| CR016 - CR195 | 0,475 | 0,125 | 3,790 | 0,008 |
| CR016 - CR204 | 0,087 | 0,116 | 0,752 | 1,000 |
| CR016 - CR214 | -0,321 | 0,109 | -2,958 | 0,119 |
| CR018 - (CR027/CR139) | 0,430 | 0,105 | 4,092 | 0,002 |
| CR018 - CR063 | 1,467 | 0,137 | 10,705 | 0,000 |
| CR018 - CR127 | 0,638 | 0,110 | 5,818 | 0,000 |
| CR018 - CR128 | 1,946 | 0,162 | 12,025 | 0,000 |
| CR018 - CR138 | 0,114 | 0,100 | 1,147 | 1,000 |
| CR018 - (CR153/CR198) | 1,883 | 0,158 | 11,911 | 0,000 |
| CR018 - CR180 | 0,981 | 0,119 | 8,245 | 0,000 |
| CR018 - CR191 | 1,883 | 0,158 | 11,911 | 0,000 |
| CR018 - (CR192/CR228) | 1,068 | 0,122 | 8,771 | 0,000 |
| CR018 - CR195 | 0,963 | 0,118 | 8,128 | 0,000 |
| CR018 - CR204 | 0,575 | 0,108 | 5,310 | 0,000 |
| CR018 - CR214 | 0,167 | 0,101 | 1,657 | 1,000 |
| (CR027/CR139) - CR063 | 1,037 | 0,142 | 7,293 | 0,000 |
| (CR027/CR139) - CR127 | 0,208 | 0,116 | 1,791 | 1,000 |
| (CR027/CR139) - CR128 | 1,515 | 0,166 | 9,121 | 0,000 |
| (CR027/CR139) - CR138 | -0,316 | 0,107 | -2,962 | 0,119 |
| (CR027/CR139) - (CR153/CR198) | 1,453 | 0,163 | 8,939 | 0,000 |
| (CR027/CR139) - CR180 | 0,551 | 0,125 | 4,411 | 0,001 |
| (CR027/CR139) - CR191 | 1,453 | 0,163 | 8,939 | 0,000 |
| (CR027/CR139) - (CR192/CR228) | 0,638 | 0,127 | 5,002 | 0,000 |
| (CR027/CR139) - CR195 | 0,532 | 0,124 | 4,281 | 0,001 |
| (CR027/CR139) - CR204 | 0,144 | 0,115 | 1,259 | 1,000 |
| (CR027/CR139) - CR214 | -0,264 | 0,107 | -2,456 | 0,449 |
| CR063 - CR127 | -0,829 | 0,145 | -5,698 | 0,000 |
| CR063 - CR128 | 0,479 | 0,188 | 2,548 | 0,371 |
| CR063 - CR138 | -1,353 | 0,138 | -9,789 | 0,000 |
| CR063 - (CR153/CR198) | 0,416 | 0,185 | 2,254 | 0,701 |
| CR063 - CR180 | -0,486 | 0,153 | -3,185 | 0,061 |
| CR063 - CR191 | 0,416 | 0,185 | 2,254 | 0,701 |
| CR063 - (CR192/CR228) | -0,399 | 0,155 | -2,578 | 0,357 |
| CR063 - CR195 | -0,504 | 0,152 | -3,316 | 0,042 |
| CR063 - CR204 | -0,892 | 0,144 | -6,180 | 0,000 |
| CR063 - CR214 | -1,300 | 0,139 | -9,373 | 0,000 |
| CR127 - CR128 | 1,308 | 0,169 | 7,737 | 0,000 |
| CR127 - CR138 | -0,524 | 0,111 | -4,713 | 0,000 |
| CR127 - (CR153/CR198) | 1,245 | 0,165 | 7,525 | 0,000 |
| CR127 - CR180 | 0,343 | 0,129 | 2,666 | 0,284 |
| CR127 - CR191 | 1,245 | 0,165 | 7,525 | 0,000 |
| CR127 - (CR192/CR228) | 0,430 | 0,131 | 3,277 | 0,047 |
| CR127 - CR195 | 0,324 | 0,128 | 2,532 | 0,374 |
| CR127 - CR204 | -0,063 | 0,119 | -0,534 | 1,000 |
| CR127 - CR214 | -0,472 | 0,112 | -4,217 | 0,001 |
| CR128 - CR138 | -1,831 | 0,163 | -11,251 | 0,000 |
| CR128 - (CR153/CR198) | -0,062 | 0,204 | -0,306 | 1,000 |
| CR128 - CR180 | -0,965 | 0,175 | -5,507 | 0,000 |
| CR128 - CR191 | -0,062 | 0,204 | -0,306 | 1,000 |
| CR128 - (CR192/CR228) | -0,878 | 0,177 | -4,957 | 0,000 |
| CR128 - CR195 | -0,983 | 0,175 | -5,625 | 0,000 |
| CR128 - CR204 | -1,371 | 0,168 | -8,158 | 0,000 |
| CR128 - CR214 | -1,779 | 0,163 | -10,898 | 0,000 |
| CR138 - (CR153/CR198) | 1,769 | 0,159 | 11,118 | 0,000 |
| CR138 - CR180 | 0,867 | 0,120 | 7,204 | 0,000 |
| CR138 - CR191 | 1,769 | 0,159 | 11,118 | 0,000 |
| CR138 - (CR192/CR228) | 0,954 | 0,123 | 7,749 | 0,000 |
| CR138 - CR195 | 0,848 | 0,120 | 7,083 | 0,000 |
| CR138 - CR204 | 0,460 | 0,110 | 4,196 | 0,002 |
| CR138 - CR214 | 0,052 | 0,102 | 0,511 | 1,000 |
| (CR153/CR198) - CR180 | -0,902 | 0,172 | -5,253 | 0,000 |
| (CR153/CR198) - CR191 | 0,000 | 0,201 | 0,000 | 1,000 |
| (CR153/CR198) - (CR192/CR228) | -0,816 | 0,174 | -4,695 | 0,000 |
| (CR153/CR198) - CR195 | -0,921 | 0,171 | -5,374 | 0,000 |
| (CR153/CR198) - CR204 | -1,309 | 0,165 | -7,955 | 0,000 |
| (CR153/CR198) - CR214 | -1,717 | 0,160 | -10,757 | 0,000 |
| CR180 - CR191 | 0,902 | 0,172 | 5,253 | 0,000 |
| CR180 - (CR192/CR228) | 0,087 | 0,139 | 0,625 | 1,000 |
| CR180 - CR195 | -0,019 | 0,136 | -0,136 | 1,000 |
| CR180 - CR204 | -0,406 | 0,127 | -3,190 | 0,061 |
| CR180 - CR214 | -0,814 | 0,121 | -6,734 | 0,000 |
| CR191 - (CR192/CR228) | -0,816 | 0,174 | -4,695 | 0,000 |
| CR191 - CR195 | -0,921 | 0,171 | -5,374 | 0,000 |
| CR191 - CR204 | -1,309 | 0,165 | -7,955 | 0,000 |
| CR191 - CR214 | -1,717 | 0,160 | -10,757 | 0,000 |
| (CR192/CR228) - CR195 | -0,105 | 0,139 | -0,761 | 1,000 |
| (CR192/CR228) - CR204 | -0,493 | 0,130 | -3,795 | 0,008 |
| (CR192/CR228) - CR214 | -0,901 | 0,124 | -7,288 | 0,000 |
| CR195 - CR204 | -0,388 | 0,127 | -3,057 | 0,092 |
| CR195 - CR214 | -0,796 | 0,120 | -6,611 | 0,000 |
| CR204 - CR214 | -0,408 | 0,110 | -3,697 | 0,011 |

Appendix S3_References

Güldal, S., 2021. The Effect of Scoring Factor for Leiden Algorithm. *Afyon Kocatepe University Journal of Sciences and Engineering* 21, 559–564. <https://doi.org/10.35414/akufemubid.870835>

Whitehead, H., 2008a. Analyzing Animal Societies, Quantitative Methods for Vertebrate Social Analysis. University of Chicago Press. <https://doi.org/10.7208/chicago/9780226895246.001.0001>

Whitehead, H., 2008b. Precision and power in the analysis of social structure using associations. *Animal Behaviour* 75, 1093–1099. <https://doi.org/10.1016/j.anbehav.2007.08.022>

Worton, B.J., 1989. Kernel Methods for Estimating the Utilization Distribution in Home-Range Studies. *Ecology* 70, 164–168. <https://doi.org/10.2307/1938423>
